# Sparse Regularization-Based Reconstruction for Bioluminescence Tomography Using a Multilevel Adaptive Finite Element Method

**DOI:** 10.1155/2011/203537

**Published:** 2010-10-04

**Authors:** Xiaowei He, Yanbin Hou, Duofang Chen, Yuchuan Jiang, Man Shen, Junting Liu, Qitan Zhang, Jie Tian

**Affiliations:** ^1^Life Sciences Research Center, School of Life Sciences and Technology, Xidian University, Xi'an 710071, China; ^2^School of Information Sciences and Technology, Northwest University, Xi'an, Shaanxi 710069, China; ^3^Institute of Automation, Chinese Academy of Sciences, Beijing 100190, China

## Abstract

Bioluminescence tomography (BLT) is a promising tool for studying physiological and pathological processes at cellular and molecular levels. In most clinical or preclinical practices, fine discretization is needed for recovering sources with acceptable resolution when solving BLT with finite element method (FEM). Nevertheless, uniformly fine meshes would cause large dataset and overfine meshes might aggravate the ill-posedness of BLT. Additionally, accurately quantitative information of density and power has not been simultaneously obtained so far. In this paper, we present a novel multilevel sparse reconstruction method based on adaptive FEM framework. In this method, permissible source region gradually reduces with adaptive local mesh refinement. By using sparse reconstruction with *l*_1_ regularization on multilevel adaptive meshes, simultaneous recovery of density and power as well as accurate source location can be achieved. Experimental results for heterogeneous phantom and mouse atlas model demonstrate its effectiveness and potentiality in the application of quantitative BLT.

## 1. Introduction

In vivo bioluminescence imaging (BLI) is a low-cost, noninvasive, and valuable tool for studying physiological and pathological processes at cellular and molecular levels. This technology has been applied to various biological models to diagnose disease, monitor therapies, and facilitate drug development [1–5]. However, due to the highly diffusive nature of the photon propagation in tissue, it is difficult to recover the depth information accurately from a planar image. To address the shortcomings of BLI, bioluminescence tomography (BLT) was developed to restore the 3D distribution of interior bioluminescent source [6]. By combining multiple BLI acquisition with anatomical structure and the associated optical properties, BLT attempts to estimate the source distributions inside a small animal with a reconstruction algorithm from the signal detected on the external surface [7].

Mathematically, BLT is a severely underdetermined and ill-posed problem, which is mainly caused by insufficient measurement and the highly diffusive nature of the photon propagation in tissue [8, 9]. There are two commonly used approaches to deal with this problem: (a) msultispectral measurement can enhance the stability of the solution by increasing the measurement information [10–12]; (b) Permissible source region (PSR) is incorporated to regularize the problem by restricting the source distribution within a local region [7, 13, 14]. The existing studies have indicated that the smaller the PSR is, the more accurate source position and power can be obtained [7]. However a small PSR is not available in most cases. For this reason, a series of reconstructions performed within progressively reduced PSRs should be a feasible way to improve result of BLT.

From a computational perspective, the challenge in BLT, as in many other imaging modalities, is to reach the desirable resolution within acceptable computational cost. As an effective numerical method, finite element method (FEM) has been widely used in BLT reconstruction especially when the domain is arbitrary geometry [7, 8, 10–14]. When solving BLT with FEM, the quality of BLT reconstruction depends on the discretization of the support domain. Generally, the finer the discretized mesh is, the better the spatial resolution. However, over-fine mesh may exacerbate the ill-posedness of the BLT inverse problem and increase the computational cost in the meantime. Hence, in [15, 16], adaptive finite element method was introduced to BLT reconstructions. Numerical simulations with regular phantom suggest that, compared with the globally uniform discretization, adaptive methods can reduce the data size and improve the computational efficiency. However, all the previous adaptive reconstruction algorithms adopt *l*_2_ norm regularization, which tends to yield nonsparse solutions. In order to obtain a satisfactory result, threshold approach is typically used to remove those artificialities caused by *l*_2_ regularization [15, 17]. 

Additionally, quantitative evaluation of the reconstructed density and power as well as accurate location is necessary in clinical or preclinical practice. For example, reconstructed total power can reflect the total tumor cell number, which is the basis for continuous monitoring but has gained less attention in most existing BLT studies so far. Especially, accurate quantitative information of density and power has not been simultaneously obtained so far. Note that the recovered density and power are associated with not only the mesh discretizations but also the regularization method used in the reconstruction. Because of the smooth characteristic of an *l*_2_ regularized solution, it is difficult to yield superior density and power simultaneously with uniformly fine meshes [16].

In the past few years, sparse regularization has been investigated in the area of compressed sensing (CS) for signal and image processing. According to the theory of CS, one can reconstruct a sparse or compressible signal from far fewer samples or measurements than what the Nyquist sampling theorem demands [18]. Recently, this technique has been introduced to enhance numerical stability and efficiency with different photon propagation models in [19–21]. Preliminary results on regular phantom show the merits and potential of CS in the application of BLT.

In this paper, inspired by CS, a novel sparse reconstruction method is proposed based on multilevel adaptive finite element framework for BLT. During the reconstruction process, the PSRs gradually shrink with adaptive local mesh refinement, which can effectively reduce the ill-posedness of BLT. In view of the characteristic of sparseness and undersampling in most BLT scenario, sparse regularization with *l*_1_ norm contributes to the enhancement in spatial resolution and algorithm stability. Numerical phantom and digital mouse atlas model are employed to validate and evaluate the performance of the proposed multilevel *l*_1_-regularized reconstruction method. 

In Section 2, the diffusion approximation of photon propagation and its finite element solution are first introduced. Then the formulation of the linear model and the multilevel *l*_1_-regularized adaptive FEM method are presented. In Section 3, numerical simulations are shown. Finally, we present the discussions and conclusion in Section 4.

## 2. Methods

### 2.1. Photon Propagation Model

The radiative transfer equation (RTE) is regarded as the most accurate model for the light transport in tissue [19]. However, RTE is computationally inefficient for practical application. Given the dominance of scattering over absorption for bioluminescent photon, diffusion equation complemented by Robin boundary condition can provide accurate description of the photon propagation in tissue [7], which is expressed as

(1) $$\begin{array}{c} -\nabla \cdot \left(D\left(r\right)\nabla \Phi \left(r\right)\right)+\mu_{a}\left(r\right)\Phi \left(r\right)=S\left(r\right)\left(r\in \Omega \right), \end{array}$$

(2) $$\begin{array}{c} \Phi \left(r\right)+2D\left(r\right)G\left(r\right)\left(v\left(r\right)\cdot \nabla \Phi \left(r\right)\right)=0\left(r\in \partial \Omega \right), \end{array}$$

where **r** ∈ *R*^3^ is the position vector in domain *Ω*, *S*(**r**) represents the power density of internal bioluminescence source, Φ(**r**) denotes the photon fluence rate, *ν* denotes the unit outer normal at boundary ∂*Ω*, *G*(**r**) is the internal reflection parameter at the boundary, *D*(**r**) = 1/(3(*μ*_*a*_(**r**) + *μ*_*s*_′(**r**))) is the optical diffusion coefficient, with *μ*_*a*_(**r**) being the optical absorption coefficient and *μ*_*s*_′(**r**) being the reduced scattering coefficient, respectively.

### 2.2. Generation of the Linear Model with FEM

As a powerful tool, FEM has been widely used for solving diffusion equations, especially for solving domain with arbitrary geometries [20]. Assuming that the optical properties of the underlying medium are given, a matrix equation that connects the discretized fluence rate Φ and the discretized source distribution *S* can be obtained with FEM [7]:

(3) MΦ=FS,

where *M* is a positive definite matrix, and *F* is the source weight matrix. Thus, the photon fluence rate Φ is derived by

(4) $$\Phi =M^{-1}FS=BS.$$

Note that only partial photon on the boundary can be captured in BLT experiments Φ is therefore partitioned into the measurable boundary data Φ^*m*^ and other immeasurable Φ^*u*^.

According to the surface photon distribution and anatomical information, the PSR can be identified as *a priori *information to restrict the reconstructed domain, thus only the source density *S*^*p*^ in PSR is taken into account. By removing the rows associated with Φ^*u*^ and retaining those rows corresponding to *S*^*p*^ in the coefficient matrix *B*, the following linear relationship is formed as follows:

(5) $$AS^{p}=\Phi^{m},$$

where *A* is a typical ill-conditioned matrix.

### 2.3. Multilevel Adaptive FEM Based Reconstruction with *l*_1_-Regularization

To achieve the necessary resolution within acceptable computational cost, the domain *Ω* is dynamically discretized in several levels rather than a fixed and uniformly fine mesh in the adaptive FEM based reconstruction process.

Let {Γ_1_,…, Γ_*k*_,…} be a sequence of tetrahedral-element mesh levels of the given domain *Ω*, where the mesh sequence changes from coarse to fine with the increase of *k*. In the reconstruction procedure, a linear relationship for each mesh level could be generated as

(6) $$A_{k}S_{k}=\Phi_{k}^{m}.$$

The multilevel adaptive FEM based reconstruction algorithm includes the following three steps:

(1) the given domain is discretized into a uniformly coarse mesh Γ_1_, where the PSR is specified as a priori knowledge to improve the reconstruction stability. The *l*_1_-regularized reconstruction algorithm is applied to the coarse mesh to yield a rough estimation of the solution, as will be shown below.

(2) identify the elements to be refined by threshold of the solution on the current mesh, and interpolate the local region to the next finer mesh. Thus, a shrunk PSR is obtained by such a local mesh refinement.

Ideally, we should conduct the mesh refinement based on rigorously derived error estimates [21]. In practice, the decision can be made by simple threshold operations of the previous solution with respect to the maximal values, that is, the elements with greater reconstructed value are selected to be refined [15, 17]. In this work, the elements with the average value of the four vertices that is no less than 20% of the maximum value are selected for refinement each time. The corresponding boundary elements are also selected to be refined. After the elements are specified, the local mesh is refined by dividing the tetrahedral element to second generation elements with the longest refinement method.

When switching a coarser mesh Γ_*k*_ to a finer mesh Γ_*k*+1_, the initial value *S*_*k*+1_^0^ of the (*k* + 1)th (*k* ≥ 1) mesh level inherits the found solution *S*_*k*_of the *k*th mesh level by linear interpolation. The nodal values outside the PSR are each set to zero;

(3) a subsequent reconstruction procedure is carried out on the new refined mesh until the stopping criteria are satisfied. We use the maximum mesh level *k*_max _ and the discrepancy between computational boundary nodal flux data Φ^*c*^ and the measured data Φ^*m*^ as the stopping criteria of the multilevel *l*_1_-regularized reconstruction procedure.

Note that, the reconstructed result at the previous mesh level not only guides mesh refinement and provides an initial value for the refined mesh, but also identifies the PSR for the subsequent reconstruction. Thus, the preliminary solution on the initial coarse mesh is very important. 

For each mesh level, BLT reconstruction is carried out by solving problem (6). In the literature, Tikhonov regularization is typically used to stabilize such problem and single out a meaningful solution by converting (6) into an optimization problem [22, 23]:

(7) $$\min\;\Theta_{k}\left(S_{k}^{p}\right)=\left\{{||A_{k}S_{k}^{p}-\Phi_{k}^{m}||}_{2}^{2}+\alpha {||S_{k}^{p}||}_{2}^{2}\right\},$$

where ||·||_2_ denotes *l*_2_ norm, and *α* is a regularization parameter.

However, due to the inherent characteristic of *l*_2_ norm, the Tikhonov regularized solution is generally nonsparse. Considering the practice of *in vivo* BLT studies, the interior bioluminescence source would have a sparse distribution. Based on CS theory, *l*_1_ regularization is a natural choice for finding out an approximately sparse solution [24]. Thereby the objective function at the *k*th level can be reformulated with *l*_1_ regularization.

(8) $$\min\;\;\Theta_{k}\left(S_{k}^{p}\right)=\left\{\frac{1}{2}{||A_{k}S_{k}^{p}-\Phi_{k}^{m}||}_{2}^{2}+\lambda {||S_{k}^{p}||}_{1}\right\},$$

where ||*S*_*k*_^*p*^||_1_ = ∑_1≤*i*≤*N*^*p*^_|*s*_*k*_^*i*^| denotes the *l*_1_ norm of the solution in PSR at the *k*th mesh level, and *λ* > 0 is the regularization parameter.

The objective function in (8) is convex but not differentiable, so solving it is more of a computational challenge than solving (7). However the simulation results in next sections will show the improvement of *l*_1_ regularization over *l*_2_ regularization in terms of localization and stability in sparse source case.

In this work, a truncated Newton interior-point method (TNIPM) is adopted at each mesh level to solve (8) [25]. The TNIPM is based on the following Lagrange dual problem of (8):

(9) $$\begin{array}{c} \max\;\;G\left(\nu \right)=-\left(\frac{1}{2}\right)\nu^{T}\nu -\nu^{T}\Phi_{k}^{m}\\ \text{s}.\text{t}.\;\;\left|{\left({A_{k}}^{T}\nu \right)}_{i}\right|\leq \lambda_{i},\;i=1,...,M. \end{array}$$

The dual problem (9) is a convex optimization problem with variable *ν* ∈ *R*^*M*^. We say *ν* is dual feasible if it satisfies the constraints of (9). According to the property of (8), from an arbitrary *S*_*k*_^*p*^, we can derive an easily computed bound on the suboptimality of *S*_*k*_^*p*^, by constructing a dual feasible point

(10) $$\begin{array}{c} \nu =a\left(A_{k}S_{k}^{p}-\Phi_{k}^{m}\right),\\ a=\min\;\;\left\{\frac{\lambda }{\left|{\left({A_{k}}^{T}A_{k}S_{k}^{p}\right)}_{i}-{\left(\Phi_{k}^{m}\right)}_{i}\right|},\;i=1,...,M\right\}. \end{array}$$

We can thus define the duality gap

(11) $$\gamma =\frac{1}{2}{||A_{k}S_{k}^{p}-\Phi_{k}^{m}||}_{2}^{2}+\lambda {||S_{k}^{p}||}_{1}-G\left(\nu \right).$$

It is obviously that the duality gap is always nonnegative, and at an optimal point, the duality gap is zero.

In TNIPM, the *l*_1_-regularized least squares problem (8) is recast to a convex quadratic problem with linear inequality constraints:

(12) $$\begin{array}{c} \min\;\;\Theta_{k}\left(S_{k}^{p},u\right)=\left\{\frac{1}{2}{||A_{k}S_{k}^{p}-\Phi_{k}^{m}||}_{2}^{2}+\lambda \overset{N^{P}}{\underset{i=1}{\sum }}u_{i}\right\},\\ \text{s}.\text{t}.\;\left|s_{i}\right|<u_{i},\;i=1,\ldots ,N^{p}, \end{array}$$

where *u* ∈ *R*^*N*^*p*^^,*N*^*p*^ is the number of nodes in PSR.

And then, adding the constraints into the minimization problem (12) by a logarithmic barrier function, the objective function of the optimization is transformed to a differentiable unconstrained problem;

(13) $$\begin{array}{cc} \min\;\;\Theta_{k}\left(S_{k}^{p}\right) & =\{\frac{1}{2}{||A_{k}S_{k}^{p}-\Phi_{k}^{m}||}_{2}^{2}+\lambda \overset{n}{\underset{i=1}{\sum }}u_{i}\\ & \;\;-\frac{1}{t}\overset{N^{p}}{\underset{i=1}{\sum }}\left[\log\;\;\left(u_{i}+s_{i}\right)+\log\;\;\left(u_{i}-s_{i}\right)\right]\}, \end{array}$$

where parameter *t* ∈ (0, *∞*). Next, we solve a sequence of (13) with increasing *t*. The detailed TNIPM algorithm for BLT reconstruction at the *k*th mesh level is presented in Algorithm 1, in which a preconditioned conjugate gradient method is adopted to compute the search direction as an approximate solution to the Newton system

(14) $$H\left[\begin{array}{c} \Delta S_{k}^{p}\\ \Delta u \end{array}\right]=-g,$$

where *H* = ∇^2^ is the Hessian, and *g* is the gradient at the current iteration [25].

As suggested by [25], we make the choice of *μ* = 2, *a*_min _ = 0.5 in the implementation. 

At the end of this subsection, we summarize the multilevel *l*_1_-regularized reconstruction algorithm in Algorithm 2.

## 3. Experiments and Results

We conducted a set of experiments with a numerical phantom model and a digital mouse model to validate the proposed multilevel *l*_1_-regularized reconstruction method. In this section, all the regularization parameters used in reconstruction were manually optimized.

The qualities of the reconstruction are quantitatively assessed in terms of location error, relative error (RE) of source density and power. Here, the reconstructed power is estimated by computing the integral of the source density over its support domain, and the corresponding RE of density and power are calculated by |*S*_recons._ − *S*_real_|/*S*_real_ and |Power_recons._ − Power_real_|/Power_real_, respectively.

### 3.1. Heterogeneous Phantom Validations

**Figure 1:**
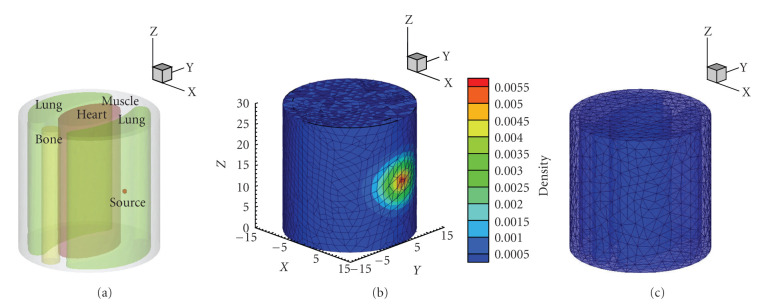
(a) Mouse chest phantom composed of muscle, lungs, heart, and bone, with one source in right lung. (b) The forward discretized mesh and the photon distribution on the surface. (c) The initial mesh used in the adaptive reconstruction, with average edge size 1.637 mm.

A cylindrical mouse chest phantom with 30 mm diameter and 30 mm height was employed to evaluate the performance of the *l*_1_-regularized multilevel AFE method. The structure of the heterogeneous phantom is shown in Figure 1(a). The specific optical properties of different organs were set as follows: *μ*_*a*_ = 0.007 mm^−1^ and *μ*_*s*_′ = 1.031 mm^−1^for muscle, *μ*_*a*_ = 0.023 mm^−1^ and *μ*_*s*_′ = 2 mm^−1^ for lung, *μ*_*a*_ = 0.011 mm^−1^ and *μ*_*s*_′ = 1.096 mm^−1^ for heart, *μ*_*a*_ = 0.001 mm^−1^ and *μ*_*s*_′ = 0.060 mm^−1^ for bone [26].

In the simulations, the phantom was discretized into a fine tetrahedral-element mesh to generate the synthetic measurements on the surface using FEM. To simulate the noise involved in real BLT experiment, 10% random Gaussian noise was added to synthetic measurements.

#### 3.1.1. Quantitative Reconstruction in Single-Source Case

Firstly, reconstruction for a single source target was attempted. A solid spherical source with 0.5 mm radius was centered at (9.5 mm, 1 mm, 15 mm) inside the right lung. The initial power source was 0.5236 nano-Watts, and the power density was 1 nano-Watts/mm^3^. The forward mesh of the phantom consisted of 11288 nodes and 62069 tetrahedral elements with 10832 boundary elements. Figure 1(b) shows the forward mesh and the photon distribution on the surface. In the multilevel reconstruction procedure, the initial coarse mesh contained 3623 nodes and 18526 tetrahedral elements, as shown in Figure 1(c), which was rather different from the forward mesh.

PSR strategy was incorporated to the reconstruction algorithm to decrease the ill-posedness of BLT. As *a priori* information of BLT reconstruction, the initial PSR was defined as {(*x*, *y*, *z*)  8 < (*x*^2^ + *y*^2^)^1/2^ < 12, 13.5 < *z* < 16.5} [26]. The subsequent PSR of the next level was identified based on the reconstruction result at the current mesh level.

**Figure 2:**
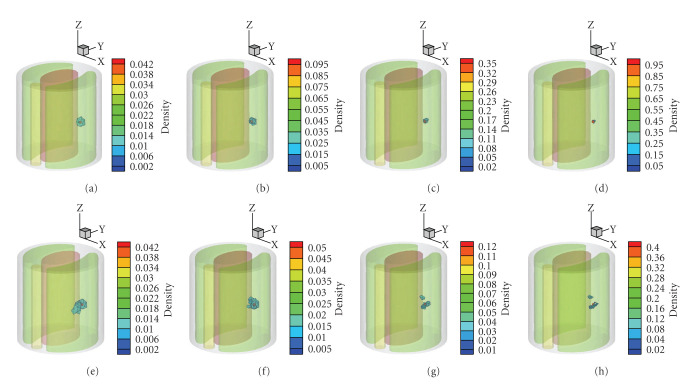
Reconstruction results in single source case on different mesh level, where the actual source is drawn as a red sphere. (a)–(d) are the isosurface of the reconstructed density by regularization from initial level to the final level, respectively. (e)–(h) are the corresponding results by regularization method, with a threshold of 50% of the maximum value.

The reconstruction was carried out using the proposed algorithm. The maximum mesh level was set to 4. The reconstructed results with regularization on multilevel adaptive meshes are shown in Figures 2(a)–2(d). For comparison, Figures 2(e)–2(h) present the reconstructed results using method, where a threshold of 50% of the maximum value was used to remove those artificialities caused by *l*_2_ regularization. The quantitative results in single source case are summarized in Table 1 in detail.

**Figure 3:**
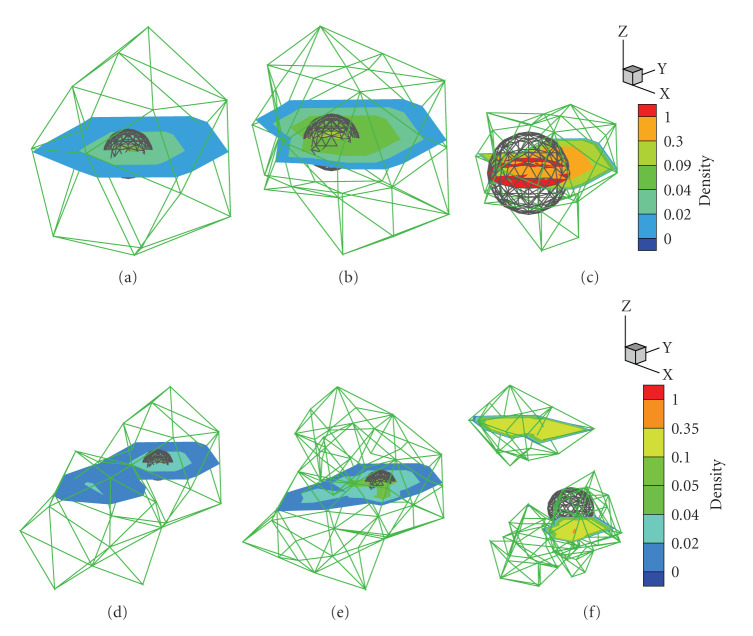
Mesh evolution in the single source case and the regularized solutions on different mesh levels. The green mesh denotes the local region around the regularized solution in PSR; the black sphere is the actual source. (a), (b), and (c) are the reconstruction by *l*_1_ regularization in the first, the second, and the last level, respectively. (d), (e) and (f) are the corresponding results by *l*_2_ regularization.

The reconstructed source positions by *l*_1_ regularization at different mesh levels stay at (9.42 mm,1.24 mm,15.02 mm), with a location error of 0.25 mm. By the adaptive mesh refinement scheme introduced in Section 2.3, the average edge size in PSR reduces during the mesh refinement. Specifically, 1.64 mm on the initial coarse mesh descends to 0.62 mm on the final mesh. The mesh evolution in multilevel reconstruction process and the reconstructed source is shown in Figure 3. 

It is noted that the quantitative information of source density and power is remarkably enhanced as the mesh became finer due to the multilevel meshes strategy. The final REs of density and power in *l*_1_ results are 0.56% and 10.94%, respectively. In the reconstruction procedure with *l*_2_ regularization, the location error is up to 1.24 mm at the initial coarse level. Despite the fact that the position and shape of reconstructed source with *l*_2_ regularization are improved with the mesh refinement, the final deviations of density and power from the initial values are comparatively bigger than those of *l*_1_ results.

**Figure 4:**
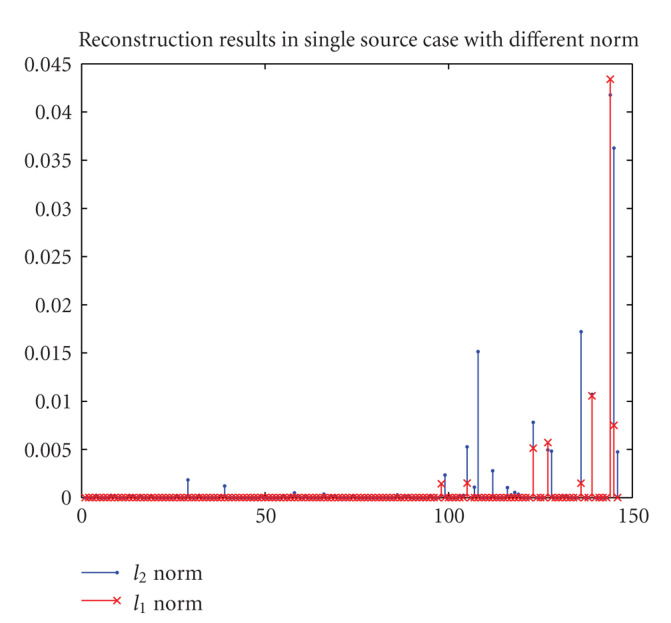
Comparison of the regularized solutions on the initial coarse mesh.

As aforementioned, compared with *l*_1_ regularization, *l*_2_ regularization tends to yield a nonsparse solution, which is demonstrated in Figure 4 by the comparison of the results on the initial coarse mesh. Furthermore, *l*_1_ regularization method provides a better initial localization than *l*_2_ does at the first mesh level, it thus yields a superior final reconstruction result to that of *l*_2_ method.

#### 3.1.2. Spatial Resolution Evaluations in Multisource Case

In order to investigate the spatial resolution capability of the proposed multilevel reconstruction method, we performed a multisource simulation experiment. Beside the spherical source located in right lung, two spatially close sources were added to the previous phantom with their centers at (−9 mm, −1.5 mm, 15 mm) and (−9 mm, 1.5 mm, 15 mm), respectively. The two sources located in left lung were 2 mm apart. The size, density, and power of each source were the same as in the single source case. The initial PSR was-same those that of single source case in this experiment. The final quantitative reconstruction results and the comparison with the actual sources are summarized in Table 2.

**Figure 5:**
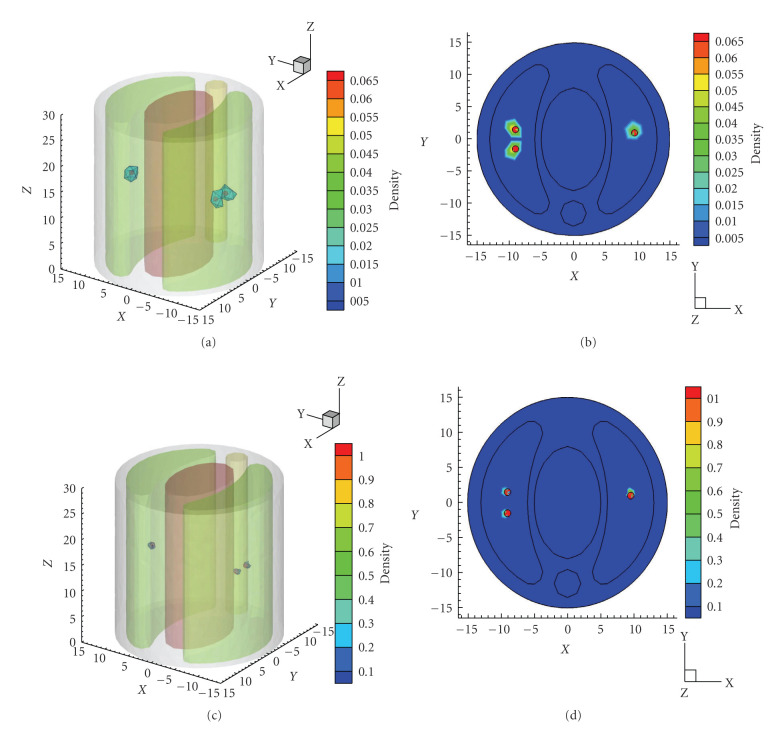
Reconstruction results in multiple sources case. (a)–(c) The isosurface of the reconstruction by the proposed method on the first and the last level, respectively. (b)–(d), The corresponding transverse view of the reconstruction at *z* = 15 mm, where the small black circles indicate the real sources.

Incorporating PSR into the reconstruction algorithm, the proposed method can always accurately distinguish these sources at different mesh levels. The reconstruction results in Figure 5 witness a remarkable improvement by the adaptive mesh refinement. During the multilevel reconstruction process, the reconstructed densities at the first mesh level are comparatively lower, and the average RE of power reaches 34.43%; with the mesh evolution, the average RE of source power falls to 11.41%. But the positions of the three sources are accurately identified on the initial coarse mesh by *l*_1_ regularization method, which lays a good foundation for the subsequent reconstruction. The figures in Table 2 demonstrate that the multilevel *l*_1_-regularized reconstruction method can provide very satisfied results in terms of spatial resolution and quantitative information about the sources.

### 3.2. 3D Digital Mouse Atlas Model Validations

**Figure 6:**
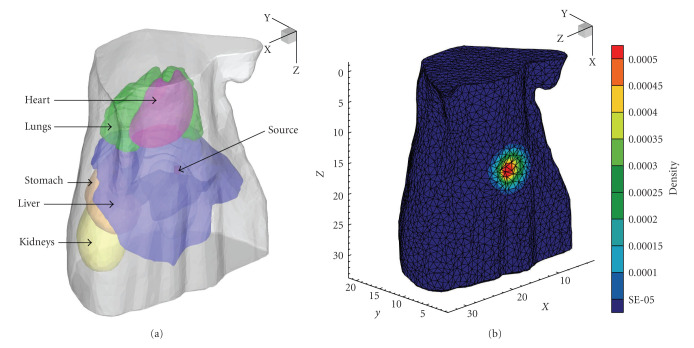
A 3D digital mouse model. (a) The torso of the mouse model with a cylindrical source in the liver. (b) Forward mesh and photon distribution on surface.

The numerical experiment with a 3D digital mouse atlas was also performed to further demonstrate the performance of the proposed reconstruction method on a real animal-shaped model. A mouse atlas of CT and cryoSection data was employed to provide anatomical information [27]. The optical properties of different organs were listed in Table 3 [28, 29]. In our simulations, the torso of the model with a height of 32 mm was chosen as the region to be investigated. A cylindrical source with 0.5 mm radius and 1 mm height was set in the liver with the center at (18.1 mm, 6.3 mm, 15.4 mm), as shown in Figure 6(a). The actual source power and density were 0.785 nano-Watts and 1 nano-Watts/mm^3^, respectively.

This torso model was discretized into tetrahedral-element mesh to generate the synthetic measurements on the boundary. The forward mesh consisted of 112795 elements and 21277 nodes, as shown in Figures 6(b). The initial mesh used in the reconstruction contained 11243 tetrahedral elements and 2382 nodes. Combining the photon distribution on the torso surface and the anatomical information, we defined {(*x*, *y*, *z*) | 10 < *x* < 26,3 < *y* < 9,12 < *z* < 19, (*x*, *y*, *z*) ∈ liver} as the initial PSR.

**Figure 7:**
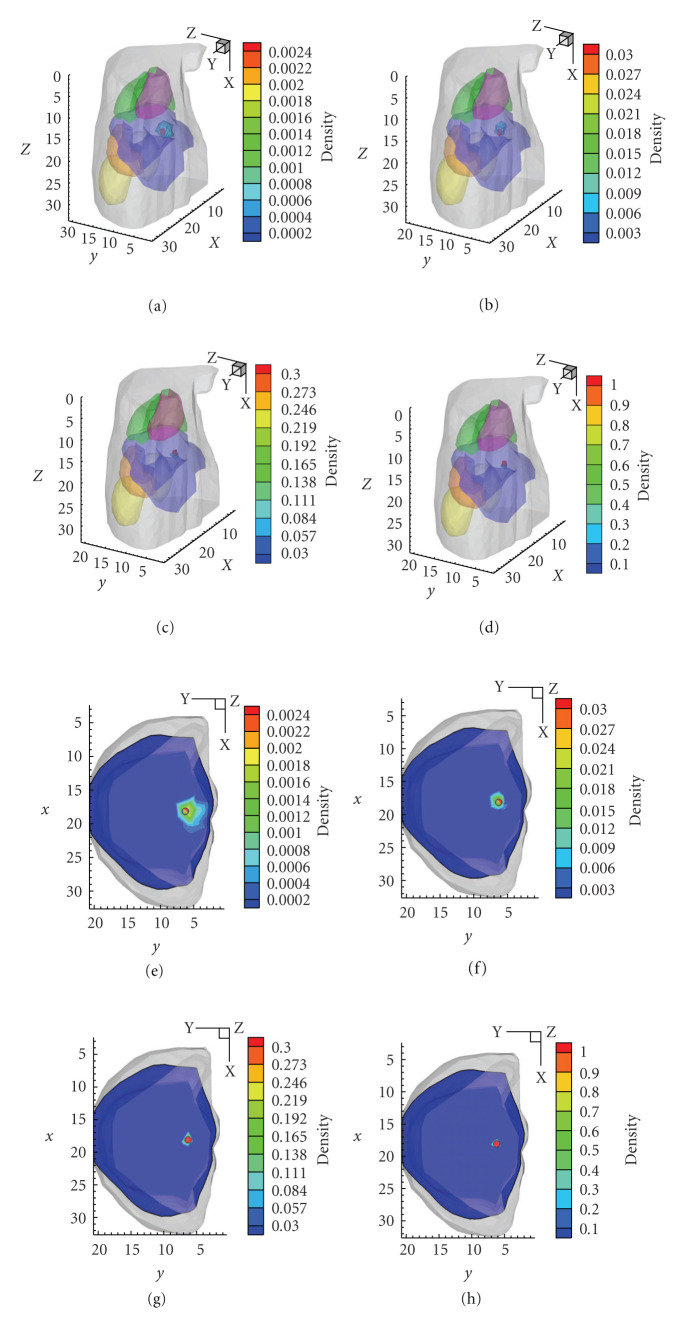
Reconstruction results of 3D digital mouse model on different mesh level. (a)–(d) are the 3D view of the results by the proposed method from the first level to the fourth level, respectively. (e)–(h) are the corresponding XY view of these results, where the small black circles indicate the real sources.

It took about 120 seconds to complete the multilevel *l*_1_-regularized reconstruction for this mouse atlas model on a laptop with Intel Pentium M processor (1.7 GHz). The detailed results on different mesh levels are given in Table 4. Due to the adaptive local mesh refinement, the mesh size in PSR reduces gradually, but the total number of nodes does not increase significantly. Although the preliminary result on the initial coarse mesh possesses relative bigger errors in source power and density, the reconstruction results are improved prominently with the mesh evolution, as shown in Figure 7 and Table 4. The final relative errors in power and density are 17.01% and 2.31%.

## 4. Discussion and Conclusion

In this paper, we present a sparse reconstruction method based on multilevel adaptive FEM and evaluated its performance in numerical simulation. Numerical simulation results suggest that the *l*_1_ regularization is effective for sparse source reconstruction. Combined with multilevel adaptive FEM, the image resolution and the quantitative information of source distribution can be remarkably enhanced.

It is well known that the density as well as position and shape of reconstructed source are significantly affected by the degree of discretization [15–17]. The existing adaptive FEM based reconstructions have demonstrated that adaptive mesh can obtain more accurate results with less computation cost compared than fixed mesh. The simulation results in Section 3 further suggest that the location and quantitative information of reconstructed source rely on not only mesh discretization but also the regularization method used in the reconstruction. 

In the existing adaptive FEM based reconstruction methods, although the source density can be remarkably improved as the mesh became finer, the reconstructed power tends to decline. The reconstruction results by using *l*_2_ regularization method in Table 1 also show this trend. The major reason to cause this phenomenon is that the smooth *l*_2_-regularized solution is commonly remedied by a big threshold.

We observed that relatively accurate power and density can be simultaneously recovered when the mesh dimension is commensurate to the source size by the proposed method. There are two key points contributing to the superior performance of the proposed reconstruction method: (1) Multilevel adaptive local mesh refinement and progressively reduced PSR can avoid the large datasets caused by uniformly fine mesh and reduce the ill-posedness of BLT, while retaining the desired accuracy in the region of interest. (2) In view of the sparsity of the source distribution, *l*_1_-regularized solution on a coarse mesh can provide a good initial localization with better numerical stability, which guides the subsequent reconstruction on finer meshes to obtain more accurate location and quantitative information of sources. 

The experiment on a mouse-shaped model with heterogeneous optical properties demonstrates the potentiality for animal experiments. Physical phantom and *in vivo* studies with the multilevel *l*_1_-regularized reconstruction method will be reported in another paper.

## Figures and Tables

**Algorithm 1:**
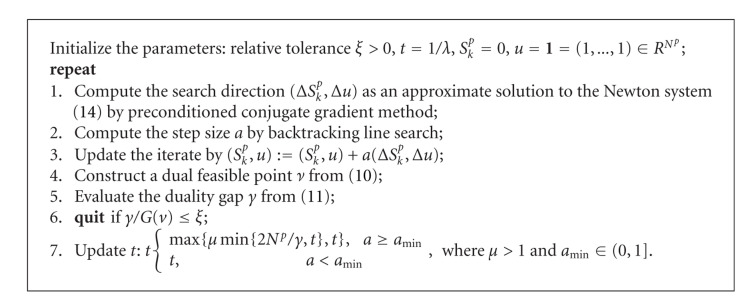
TNIPM algorithm for BLT reconstruction at the *k*th mesh level.

**Algorithm 2:**
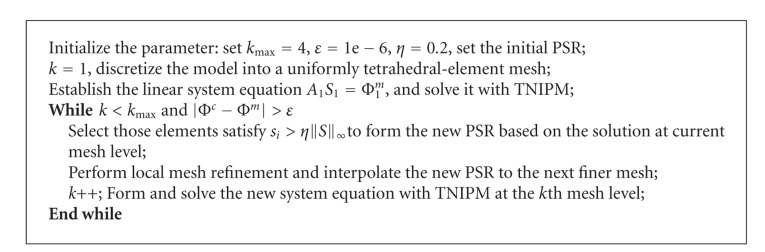
Multilevel *l*_1_-regularized reconstruction algorithm.

**Table 1:** Reconstruction results in single source case on different mesh levels.

Mesh level	Regular. method	Number of nodes	Location center	Error (mm)	Density (nW/mm^3^)	RE of density	Recon. power (nW)	RE of power
1	*l*_1_	3623	9.42,1.24,15.02	0.25	0.0434	95.66%	0.3315	36.69%
	*l*_2_	3623	9.43,−0.16,14.55	1.24	0.0418	95.82%	0.2896	44.69%
2	*l*_1_	3924	9.42,1.24,15.02	0.25	0.0942	90.58%	0.4515	13.77%
	*l*_2_	3960	9.18,0.43,15.26	0.70	0.0522	94.78%	0.3396	35.14%
3	*l*_1_	4242	9.42,1.24,15.02	0.25	0.3105	68.95%	0.4537	13.35%
	*l*_2_	4435	9.83,0.96,15.42	0.54	0.1227	87.73%	0.4185	20.07%
4	*l*_1_	4910	9.42,1.24,15.02	0.25	1.0056	5.6%	0.4663	10.94%
	*l*_2_	5232	9.40,0.54,15.26	0.54	0.3739	62.61%	0.3700	29.34%

**Table 2:** Quantitative results and the comparison with the actual sources in multisource case.

Source	Actual position	Recon. position	Location error (mm)	Recon. density (nW/mm^3^)	RE of density	Recon. power (nW)	RE of power
Source-1	(9.5,1,15)	(9.42,1.24,15.02)	0.49	1.06	6%	0.4916	6.12%
Source-2	(−9, 1.5,15)	(−9.29,1.5,15.06)	0.30	0.5713	42.87%	0.4416	15.66%
Source-3	(−9, −1.5,15)	(−9.30, −1.46,15.08)	0.31	0.6205	37.95%	0.4584	12.45%

**Table 3:** Optical properties for the atlas organs region.

Material	Muscle	Lung	Heart	Liver	Kidney	Stomach
*μ*_a_[mm^−1^]	0.23	0.35	0.11	0.45	0.12	0.21
*μ*_s_′[mm^−1^]	1	2.3	1.1	2	1.2	1.7

**Table 4:** Reconstruction results for 3D atlas model on different mesh level.

Mesh level	Mesh size In PR	Number of nodes	Location center	Error (mm)	Density (nW/mm^3^)	Power
1	1.6833	2382	(17.85,6.22,14.85)	0.61	0.0026	0.0636
2	1.3087	2641	(17.85,6.22,14.85)	0.61	0.0309	0.1951
3	0.8277	3033	(17.99,6.31,15.88)	0.49	0.3080	0.3677
4	0.6181	3908	(17.99,6.31,15.88)	0.49	1.0231	0.6518
